# The Adjunctive Role of Antimicrobial Photodynamic Therapy to Non-Surgical Treatment in Patients with Type 2 Diabetes Mellitus: A Systematic Review and Meta-Analysis

**DOI:** 10.3390/healthcare13141703

**Published:** 2025-07-15

**Authors:** Alessia Pardo, Annarita Signoriello, Elena Messina, Elia Stilo, Rachele De’ Manzoni Casarola, Elisabetta Ferrara, Giorgio Lombardo, Massimo Albanese

**Affiliations:** 1 Section of Oral and Maxillofacial Surgery, Department of Surgical Sciences, Dentistry, Gynecology and Pediatrics, University of Verona, 37124 Verona, Italy; elena.messina@univr.it (E.M.); eliastilo2001@gmail.com (E.S.); giorgio.lombardo@univr.it (G.L.); massimo.albanese@univr.it (M.A.); 2 Pediatric Dentistry and Dental Hygiene Unit, IRCCS—Sacro Cuore Hospital of Negrar, 37024 Verona, Italy; rachele.demanzonicasarola@sacrocuore.it; 3 Department of Human Sciences, Law and Economics, Telematic University Leonardo da Vinci, Torrevecchia Teatina, 66100 Chieti, Italy; elisabetta.ferrara@unidav.it

**Keywords:** periodontitis, diabetes mellitus, antimicrobial photodynamic therapy, adjunctive therapy

## Abstract

**Background:** This systematic review aimed to assess the outcomes related to the use of antimicrobial photodynamic therapy (aPDT) as an adjunct to non-surgical periodontal treatment (NSPT) of patients affected by periodontitis and with type 2 diabetes mellitus (T2DM). **Methods:** PubMed, Cochrane Library, Scopus, and Web of Science (core collection) were queried up to January 2025. The PICO question investigated the comparison between T2DM patients undergoing NSPT with or without aPDT, in terms of improvement of clinical parameters. Two independent operators performed the study selection, data extraction, and risk of bias assessment (RoB-2 tool). The meta-analysis examined the reduction in bleeding on probing (BoP) and probing pocket depth (PPD) in sites > 4 mm, reporting mean difference (MD) and 95% confidence intervals (CIs). **Results:** Among 502 studies retrieved, 15 were finally included in the systematic review and meta-analysis. In T2DM individuals, the adjunct of aPDT to NSPT demonstrated a substantial reduction in BoP and PPD after 3 and 6 months compared to the use of NSPT alone. **Conclusions:** The outcomes of this systematic review suggest that adjunctive aPDT may provide additional benefit to NSPT in reducing inflammation in T2DM patients with periodontitis, indicating that this combined therapy could represent a potentially useful approach for individuals with T2DM. Review registration: registration in PROSPERO (International prospective register of systematic reviews) with ID CRD42024506295 on 6 February 2024.

## 1. Introduction

Periodontitis is a chronic disease caused by the reaction of the host immune system to adverse subgingival Gram-negative bacterial species, which maintain inflammation in gingival and periodontal tissues [1]. The red complex of *Porphyromonas gingivalis*, *Treponema denticola*, and *Tanneralla forsythia* has been widely demonstrated to have consistent effects on the oral microbial population during periodontal disease [2], triggering a state of dysbiosis and inflammation, ultimately leading to bone loss.

Similar inflammatory mechanisms of soft tissue damage and progressive alveolar bone loss in periodontitis are also found in other chronic diseases, such as diabetes mellitus (DM) [3]. DM is a global epidemic disease characterized by metabolic derangement in the form of hyperglycemia, resulting from impaired or absent insulin secretion, insulin action, or both [4]. DM has a wide impact on health due to long-term manifestations [5], including retinopathy, neuropathy, atherosclerosis, and impaired wound healing.

Periodontal diseases are recognized as the sixth most common diabetes-related complication, and, at the same time, the most common oral problem in diabetic patients [6]. According to the bidirectional link between these conditions [7], an inflamed status can also affect glycemic control and increase cardiovascular complications of diabetes: (i) in the diabetes–periodontitis direction, hyperglycemia is associated with increased risk and severity of periodontitis and worse periodontal outcomes following periodontal therapy; (ii) in the periodontitis–diabetes direction, early diagnosis has led to the initiation of cost-effective lifestyle changes that have led to a significant portion of patients transitioning from prediabetes to normoglycemia [8].

As specific systemic conditions are considered predisposing factors for bacteria-induced inflammation, the link between DM and inflammatory response to oral pathogens currently represents a field of great interest [9]. It has been reported that diabetes mellitus has a direct influence of osteoclasts’ activity and osteoblasts’ apoptosis [10,11], while hyperglycemia can indirectly influence oral tissue inflammation through the formation of advanced glycation products and specific pro-inflammatory cytokines, which are detectable in gingival crevicular fluid both of type 1 and type 2 diabetes mellitus patients (T2DM). In this way, this cytokines’ dysregulation, associated with other molecular mechanisms, can lead to an altered host response to bacterial challenge [11]. It is, therefore, of fundamental importance [12] for diabetic patients to undergo regular dental check-ups as an integral part of diabetes care.

Recent strategies in the non-surgical treatment of periodontal diseases aim to enhance mechanical removal of plaque/biofilm deposits by combining conventional supra-gingival and subgingival instrumentations with adjunctive means [13,14]. These supplementary alternative approaches not only demonstrate a more effective reduction in bacterial deposits in sites particularly difficult to access for mechanical instruments [15] but also provide targeting of specific target components of the periodontal inflammatory response [16].

Antimicrobial therapies described in the literature offer advantages in clinical practice by preventing the potential development of bacterial resistance, a limitation associated with the use of locally delivered antibiotics [17,18,19]. Furthermore, non-responsive cases, particularly those refractory to conventional treatments, often require targeted therapeutic approaches, frequently involving modulation of inflammatory mechanisms [5].

Laser therapy demonstrates a consistent reduction in bacterial load in periodontal pockets [20], as its effects are enhanced by the absorption properties of hemoglobin, melanin, and pigmented periodontal bacteria [21]. This effect can be improved by the inclusion of a photosensitizing dye, as in adjunctive antimicrobial photodynamic therapy (aPDT). In this regard, use of aPDT in non-surgical periodontal treatment (NSPT) of persistent periodontal pockets demonstrates the advantage [13,22] of a locally administered non-invasive approach. This therapy has been recently assessed in several studies comparing it with traditional debridement techniques for the treatment of periodontitis [23,24,25], revealing clinical improvements in terms of soft tissue parameters, such as probing pocket depth (PPD), bleeding on probing (BoP), and clinical attachment level (CAL).

Despite promising outcomes reported for the use of aPDT in cases of chronic inflammatory periodontal conditions [21,22,26,27,28], also typical of T2DM, there is no uniform evidence [29] concerning the clinical and/or microbiological benefits of aPDT associated with conventional debridement, as some studies report similar results for both techniques [30,31,32,33,34]. Although periodontitis was reported to be more prevalent and severe in patients with DM than in non-diabetic patients, studies have shown that diabetic patients with good metabolic control appear to have a similar clinical and microbiological response to NSPT as patients without diabetes [8]. Furthermore, heterogeneity in study design does not provide a clear interpretation of results in terms of superior outcomes of aPDT combined with NSPT compared to NSPT alone. Taking into account the solid evidence regarding the positive influence of NSPT on T2DM patients, together with the demonstrated antimicrobial properties of aPDT but with still no general consensus on the comparison between aPDT combined with NSPT and NSPT alone, the clinical rationale of the present study specifically focused on assessing the possible enhancing effects of aPDT on NSPT in a targeted-type population already widely investigated for their correlation with periodontitis.

In light of these considerations, the focus of the present systematic review, with meta-analysis, was to explore the role of adjunctive aPDT, when associated with NSPT, in improving periodontal inflammatory parameters of PPD and BoP in a condition (T2DM) typically associated with periodontitis.

## 2. Materials and Methods

This systematic review evaluates the current scientific evidence up to January 2025 regarding the effect of aPDT in addition to NSPT in individuals with T2DM and periodontal pockets equal to or greater than 4 mm.

### 2.1. Focused Question and PICO

The specific clinical question was as follows: “Does the addition of aPDT to NSPT improve clinical parameters in individuals with T2DM and periodontitis, compared to NSPT alone?”

The PICO question was disaggregated into Patients (P), Intervention (I), Comparison (C), and Outcomes (O), as follows:

- Patients = individuals with T2DM and periodontitis;
- Intervention = aPDT combined with NSPT;
- Comparison = NSPT (alone);
- Outcome = clinical parameters.

Randomized clinical trials were included in the systematic review, involving the following:

- A test group of patients with T2DM and periodontitis, receiving both aPDT and NSPT;
- A control group of patients with T2DM and periodontitis, receiving only NSPT.

Following an initial search using major search engines, studies were excluded based on duplication criteria. Additional criteria, such as article type for clinical trials and text availability for full-text access, were also applied. Subsequently, the following were excluded: conference abstracts, editorial comments, studies lacking control groups, cross-sectional studies, case–control studies, and research project publications without results.

The selection process was conducted in accordance with the Cochrane guidelines and reported following the PRISMA (Preferred Reporting Items for Systematic Reviews and Meta-Analyses) statement [35].

The present study was registered in PROSPERO (International Prospective Register of Systematic Reviews) with ID CRD42024506295 on 6 February 2024.

### 2.2. Eligibility Criteria

The inclusion criteria were as follows:

- Randomized controlled clinical trials (RCTs);
- Patients diagnosed with periodontitis;
- Patients diagnosed with T2DM, confirmed through glycosylated hemoglobin (HbA1c) measurement;
- Number of patients per group > 15;
- Primary clinical parameters: probing pocket depth (PPD), bleeding on probing (BoP).

The exclusion criteria were as follows:

- Studies conducted on animals;
- Studies related to dental implants;
- Studies regarding the systemic and/or topical administration of antibiotics.

### 2.3. Information Sources and Search Strategy

The literature search was conducted on comprehensive electronic databases including PubMed, Cochrane Library, Scopus, and Web of Science (core collection).

The search algorithms were constructed as follows, with no restrictions on language or limitations on the publication year:

“(photodynamic OR laser) AND (periodontitis OR periodontal diseases OR periodontal disease) AND diabetes”.

### 2.4. Study Selection, Screening, and Data Collection

Two reviewers (A.P. and A.S.) independently carried out the search up until 13 January 2025, screening the titles, abstracts, and full texts of the studies obtained through the searches. They used the predetermined inclusion and exclusion criteria. Any disagreements regarding eligibility were resolved through discussions between the reviewers. Subsequently, the studies that fulfilled all the inclusion criteria were selected for data extraction. Data collection from the included reports was performed by the same first reviewers (A.P. and A.S.), who independently worked with an Excel spreadsheet. From the selected articles were evaluated, and the following data were extracted: author’s name and publication year, country where the study was conducted, sample size, duration of follow-up period, HbA1c levels of participants before and after the intervention, type of photodynamic therapy protocol used in the aPDT group, and PPD and BoP before and after the intervention for both the aPDT and NSPT group.

According to the objectives, the primary outcomes of the evaluated studies were the clinical parameters of PPD and BoP.

### 2.5. Risk of Bias Assessment and Effect Measure

The Cochrane Risk of Bias Tool for Randomized Trials [36] was employed to assess the risk of bias. Each study included in the analysis was classified into three categories based on the criteria set forth in the tool (“low risk of bias,” “some concerns regarding bias,” or “high risk of bias”). In the event of a disagreement between the two reviewers, additional discussion was used to reach a consensus.

The primary outcomes evaluated in the meta-analysis were the parameters of PPD and BoP, and the effect measures utilized in the synthesis were mean and standard deviation, assessed separately at 3 months of follow-up for BoP and at 3 and 6 months of follow-up for PPD. A secondary outcome assessed was CAL at 3 months of follow-up.

### 2.6. Data Synthesis and Certainty Assessment

Studies were eligible for the meta-analysis if they included test and control groups that reported BoP and CAL values at 3 months, and PPD values at 3 and 6 months of follow-up, and only sites with PPD greater than 4 mm. The meta-analysis was conducted using Review Manager Web [37], with a random effects model chosen based on the degree of heterogeneity. The inverse-variance method was employed, and the heterogeneity was quantified using the I^2^ statistic.

The certainty assessment was conducted using GRADEpro GDT [38]. The two reviewers evaluated the level of confidence of the evidence based on the following factors: risk of bias, inconsistency, indirectness, imprecision, publication bias, large effect, plausible confounding, and dose–response gradient. In the event of disagreement between the two reviewers, additional discussion was used to reach a consensus.

## 3. Results

### 3.1. Study Selection

**Figure 1:**
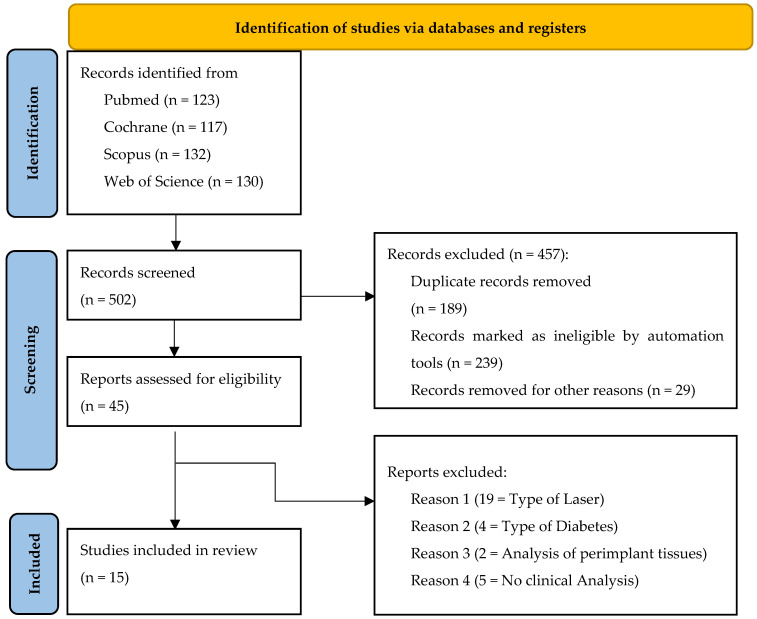
PRISMA flow diagram of the search and selection process [35].

The search was conducted until 13 January 2025. During the electronic search, a total of 502 studies were retrieved. Of these, 457 articles were excluded, including 189 duplicates, 239 that were ineligible, and 29 removed for other reasons. Forty-five studies were selected for full-text analysis. Following a thorough evaluation of the full text, 15 studies [39,40,41,42,43,44,45,46,47,48,49,50,51,52,53] were included in this systematic review and meta-analysis. The selection process is reported in a flowchart in accordance with the PRISMA guidelines (Figure 1).

### 3.2. Study Characteristics

All studies examined in the systematic review were randomized controlled trials (RCTs) that were carried out in Brazil, Saudi Arabia, Pakistan, Japan, Turkey, Romania, and Slovenia. The number of participants in each study ranged from 12 to 66. The maximum follow-up period for the samples in the studies lasted no longer than 12 months after undergoing NSPT alone, or after receiving aPDT in conjunction with NSPT: specifically, 4 studies [39,40,42,52] included a maximum three-month follow-up, 10 [41,43,49,51,53] also included a six-month follow-up, and 1 [44] included a twelve-month follow-up. All studies provided information on the glycemic status of the participants by reporting the mean values of HbA1c, ranging from 6.5% to 11%.

Among the studies evaluated, 10 [39,40,42,43,46,47,50,51,52,53] compared the test and control groups for periodontal indices. The other five studies [41,44,45,48,49] utilized a split-mouth design, comparing periodontal pockets in different quadrants with one serving as a control and the other as the test group.

All studies compared test and control groups at each time point (baseline and follow-ups), generally finding no significant differences between groups in most cases, despite an overall improvement of periodontal conditions. However, one study [49] demonstrated that combining aPDT with NSPT led to a more significant reduction in PPD and BoP, as well as a greater improvement of CAL, in well-controlled T2DM patients, both at 3- and 6-month follow-up, compared to NSPT alone. In poorly controlled T2DM patients, on the other hand, aPDT in conjunction with NSPT resulted in a greater reduction in BoP and PPD at 3 months, only in BoP at 6 months, compared to NSPT alone.

Two additional studies [51,52] found that adding aPDT to NSPT resulted in a more significant reduction in PPD and greater CAL gain at 3 and 6 months post-therapy, respectively, compared to NSPT alone.

Furthermore, two studies [39,46] compared variations in PPD between different time points within each group without inter-group comparisons, while one study did not report any statistical comparison between groups [47].

The details and outcomes of the included studies are summarized in Table 1.

### 3.3. Risk of Bias

**Figure 2:**
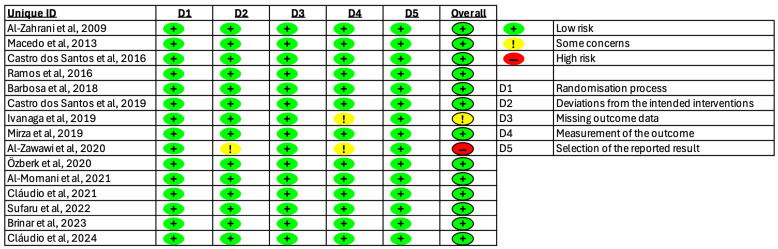
Risk of bias in the included studies.

The classification of bias risk for the included studies is depicted in Figure 2, revealing that the majority of them were identified as having “Low risk” of bias.

### 3.4. Results of Syntheses

A meta-analysis comparing the test group (aPDT + NSPT) and the control group (NSPT alone) was conducted. One study [53] was excluded from the meta-analysis because of its use of BM (active oxygen-releasing gel) in both groups.

**Figure 3:**
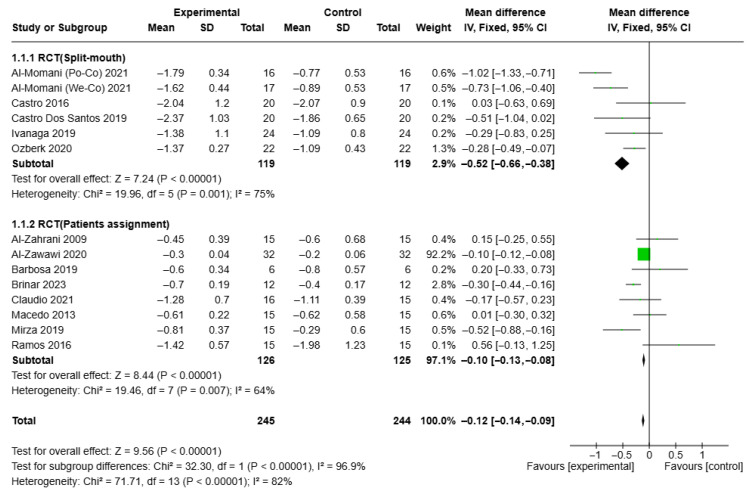
Forest plot of PPD at 3-month follow-up in aPDT + NSPT test group and NSPT control group, according to study-design sub-group analysis.

**Figure 4:**
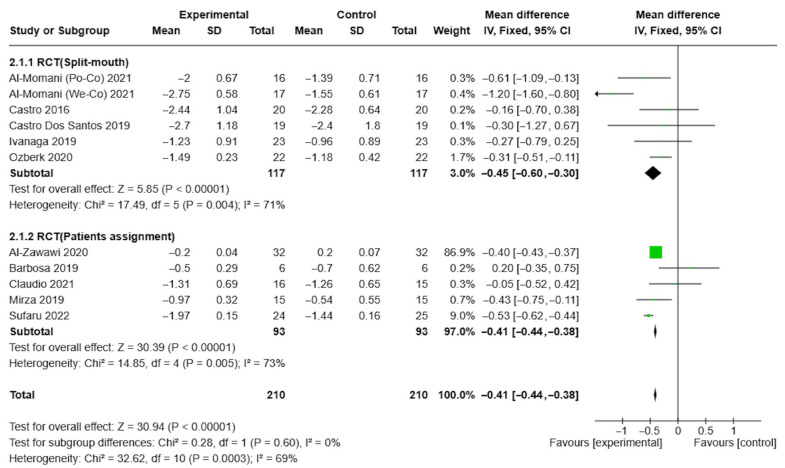
Forest plot of PPD at 6-month follow-up in aPDT + NSPT test group and NSPT control group, according to study-design sub-group analysis.

The results showed that the reduction in PPD was significantly greater in the test group at both the 3-month follow-up (MD = −0.26, CI = −0.42, −0.1, I2 = 82%) and 6-month follow-up (MD = −0.42, 95% CI = −0.54, −0.3, I2 = 69%). These findings are illustrated in the forest plots shown in Figure 3 and Figure 4.

**Figure 5:**
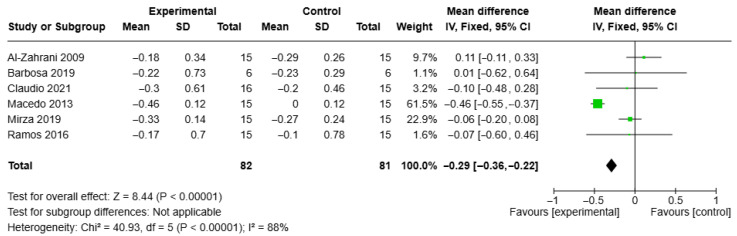
Forest plot of BoP at 3-month follow-up in aPDT + NSPT test group and NSPT control group. Study design was RCT with patients’ assignment for all studies, so sub-group analysis was not included.

The results showed that the reduction in BoP after 3 months was significantly greater in the test group (MD = −0.29, 95% CI = −0.36, −0.22, I2 = 88%). These findings are illustrated in the forest plots shown in Figure 5.

**Figure 6:**
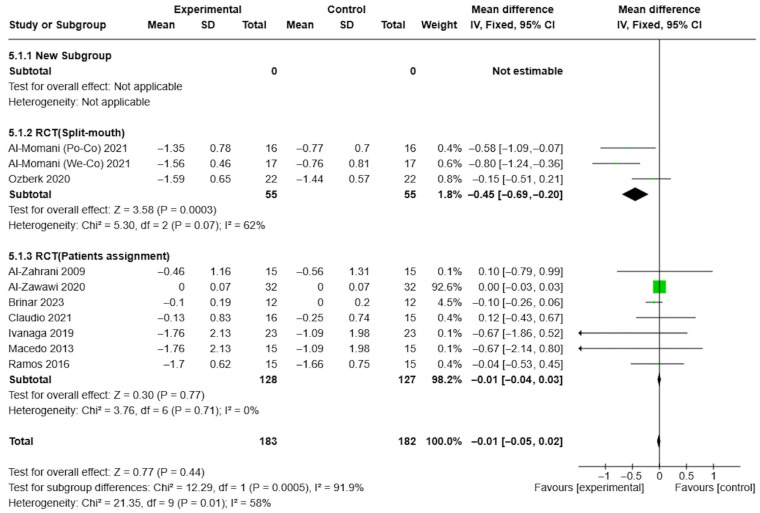
Forest plot of CAL at 3-month follow-up in aPDT + NSPT test group and NSPT control group, according to study-design sub-group analysis.

The results showed that the reduction in CAL after 3 months in the test group (MD = −0.01, 95% CI = −0.05, 0.02, I2 = 58%). These findings are illustrated in the forest plots shown in Figure 6.

### 3.5. Certainty of Evidence

**Figure 7:**
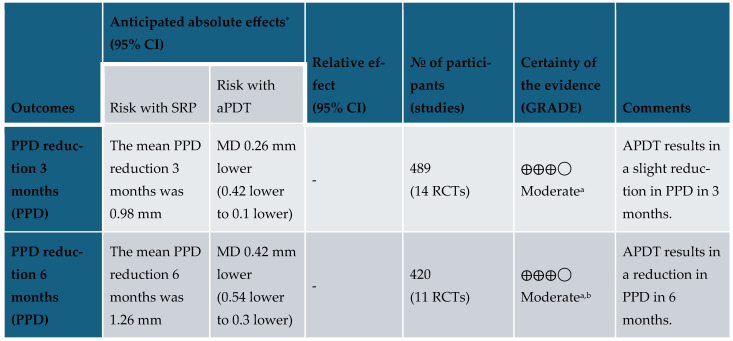
Summary of findings: * The risk in the intervention group (and its 95% confidence interval) is based on the assumed risk in the comparison group and the relative effect of the intervention (and its 95% CI); CI: confidence interval; MD: mean difference. GRADE Working Group grades of evidence: High certainty (we are very confident that the true effect lies close to that of the estimate of the effect); Moderate certainty (we are moderately confident in the effect estimate; the true effect is likely to be close to the estimate of the effect, but there is a possibility that it is substantially different); Low certainty (our confidence in the effect estimate is limited; the true effect may be substantially different from the estimate of the effect); Very low certainty (we have very little confidence in the effect estimate; the true effect is likely to be substantially different from the estimate of the effect). ^a^: Visual inconsistency and statistical analysis also showing heterogeneity; ^b^: The evidence directly answers the health care question.

According to the GRADEpro GDT [38], as depicted in Figure 6, the overall certainty of evidence was assessed as moderate, based on comparisons between the two interventions and the controls. Based on this assessment, aPDT in combination with NSPT can be recommended with caution to reduce BoP and PPD at both 3-month and 6-month follow-up (Figure 7).

## 4. Discussion

The oral microbiome plays a critical role in periodontitis, with implications for systemic health through pathogen inhibition, immune regulation, nutrient absorption, and metabolism [54]. Furthermore, oral bacteria can migrate to extraoral sites, causing infections and inflammation; alteration of the oral microbial community structure has thus been hypothesized to be linked to cardiovascular disease, stroke and Alzheimer’s disease, obesity, and diabetes.

According to current evidence, DM represents a major risk factor for periodontitis (the risk is approximately three times higher in diabetic compared to non-diabetic individuals [55]), and thus, the control of HbA1c levels is crucial in determining increased or decreased gingival and periodontal inflammation [8,56]. Moreover, several authors have reported that even the prevalence and severity of non-oral diabetes-related complications (e.g., retinopathy, diabetic neuropathy, proteinuria, and cardiovascular complications) [57,58] are correlated with the severity of periodontitis. The presence of specific periodontal pathogens causes a notable immune-inflammatory response, which is likely responsible for the greater risk and severity of periodontal disease in diabetics [59].

Similarly to other systemic conditions affecting oral health [60,61], the link between DM and periodontitis can be described as bidirectional [62]: both diseases are considered risk factors for one another, with direct consequences in case of the improvement or worsening of clinical conditions. Although type 1 DM has been shown to severely increase the risk of periodontitis, with early bone loss even in children and younger individuals [57,63], the relationship between the two diseases has been mostly investigated for type 2 DM, as the age of onset for both diseases tends to overlap [64].

Plaque accumulation and subgingival biofilm composition in periodontal pockets lead to a dysregulated secretion of host-derived mediators of inflammation and soft tissue breakdown [11,65], with associated elevated levels of systemic inflammatory markers (in both type 1 and type 2 DM). Thus, as periodontal bacteria trigger secretion of these mediators, two systemic effects can be observed: a direct induction of cell proliferation and genetic variants, which reduces the ability to adapt to oxidative stress and apoptosis [66], and indirect damage to pancreatic beta cells [67,68], which increases insulin resistance and downregulates the control of HbA1c levels.

Given this relationship, the role of periodontal treatment, especially non-surgical maintenance, is fundamental to improve clinical, immunological, and microbiological parameters for the stability of periodontal disease [8]. Several studies on diabetes patients treated with conventional periodontal therapy [69,70,71,72,73] have confirmed a positive effect on local fibroblast proliferation, together with significant reductions in serum levels of inflammatory mediators (e.g., IL-6, TNF-α), and decreased HbA1c.

The use of PDT in NSPT in T2DM patients is an emerging field of interest: when the photosensitizer undergoes a transition from a low to a higher energy state, aPDT antimicrobial properties are based on the production of cytotoxic reactive oxygen species by a photoactivatable agent (or photosensitizer) exposed to light of a compatible wavelength [13,74]. Among different photosensitizing dyes with lasers of various wavelengths, those with antibacterial properties [e.g., Indocyanine green (ICG)] are suitable for oral applications due to their low toxicity, absence of side effects, easy and safe use, absorption peak close to the emission maximum of available dental diode lasers (near 800 nm), and rapid elimination [75,76].

The controversial outcomes reported in the literature [40,41,42,43,44,45,48,50,77,78] do not allow for proper interpretation of results in terms of superior outcomes with aPDT combined with NSPT compared to NSPT alone. Based on current evidence regarding the positive influence of NSPT on T2DM patients, together with the demonstrated antimicrobial properties of aPDT, this study specifically focused on the possible enhancing effects of aPDT on NSPT in the targeted type of patients with T2DM.

All RCTs examined in this systematic review presented short-term evaluations with a maximum of 12 months after therapy, with heterogeneous follow-up, and mostly [41,43,44,45,46,47,48,49,51,53] included a six-month follow-up.

Most studies [39,40,42,43,46,47,50,51,52,53] compared the test and control groups for periodontal indices, while fewer studies utilized the split-mouth design. In addition, when comparing test and control groups at each time point (baseline and follow-ups), no significant differences between groups were found in most cases, despite an overall improvement of periodontal conditions.

Only one study [49] showed aPDT combined with NSPT as a therapy with a more significant reduction in PPD and BoP, as well as a greater CAL gain, in well-controlled T2DM patients, both at 3- and 6-month follow-up, compared to NSPT alone. Two additional studies [51,52] found that adding aPDT to NSPT resulted in more significant reductions in PPD and greater CAL gain at 3 and 6 months post-therapy, respectively, compared to NSPT alone. Furthermore, two other studies [39,46] compared variations in PPD between different time points within each group without inter-group comparisons.

Concerning the meta-analysis, aPDT added to NSPT, compared to NSPT alone, seems to offer benefits in reducing PPD and BoP, with overall improvement in periodontal conditions in individuals with T2DM undergoing periodontal treatment, particularly at 3 and 6 months post-intervention.

Specifically, three studies [49,51,52] found a significant reduction in PPD at 3 and 6 months post-therapy, respectively, compared to NSPT alone. The results showed that these reductions were significantly greater in the test group at both 3 months (MD = −0.26, 95% CI = −0.42, −0.1, I2 = 82%) and 6 months of follow-up (MD = −0.42, CI = −0.54, −0.3, I2 = 69%).

The results showed that the reduction in BoP after 3 months was significantly higher in the test group (MD = −0.29, CI = −0.36, −0.22, I2 = 88%). These findings are illustrated in the forest plots shown in Figure 5.

However, these positive outcomes must be considered with caution, as the overall certainty of evidence was assessed as moderate, based on comparisons between the two interventions and the controls. Within the limitations of this meta-analysis, PPD and BoP can be considered reliable indicators of improvement in periodontal inflammation.

Based on the above-mentioned results, aPDT is recommended in combination with NSPT to improve BoP and PPD at the 3-month and 6-month follow-up. This significant decrease represents a sign of inflammatory resolution, in line with the outcomes of previous clinical studies [24,25]. With regard to periodontal parameters, benefits in BoP reduction [8] and CAL gain [79] were found, while no significant changes were observed for crestal bone levels.

As bleeding may persist because of tooth position or other local factors limiting access for treatment and optimal plaque control [13,22,80], repeated aPDT administrations have been shown to be effective in reducing residual pockets and BoP [24], thus decreasing the perceived need of pharmacological treatment or surgery, and constituting a safe alternative to antibiotic administration in periodontal setting [81,82]. On the other hand, several authors have found comparable results with conventional therapy alone [83,84], especially regarding improvement of BoP. Additionally, considering plaque indices, as directly dependent on the patient’s compliance with oral hygiene instructions, aPDT did not show different outcomes compared to NSPT alone [34].

Considering the demonstrated contribution of periodontal treatment to reducing HbA1c levels and promoting proper glycemic control [71,72], patients of the studies included in this review mostly presented mean values of HbA1c ranging from 6.5% to 11% (below 9%) at baseline. Patients with controlled T2DM present HbA1c levels between 6.5 and 7.0% [4], receiving dietary therapy and/or hypoglycemic drugs with regular follow-up at the diabetes service. Values > 7% usually require adjustment of therapy, so this value can be considered a clinical threshold in evaluating diabetic control, which can influence oral conditions. As only one study [49] distinguished well-controlled from poorly controlled T2DM patients, no definitive conclusions can be deduced. However, it can be hypothesized that proper control can limit excessive bacterial load and inflammation, facilitating the management of patient compliance at follow-up appointments. Nevertheless, it can be stated that overall mean HbA1c levels at baseline did not apparently influence the outcomes of clinical inflammatory reduction in terms of PPD and BoP.

In light of these findings, more studies are encouraged to explore further perspectives concerning the inclusion of more clinical parameters (beyond PPD and BoP), especially for long-term follow-up of persistent deep pockets after therapy. The preliminary outcomes retrieved from this systematic review and meta-analysis also suggest promising non-invasive strategies aiming to satisfy patients’ comfort and needs.

### Study Limitations

The limitations of this systematic review and meta-analysis are mainly related to the heterogeneity of initial systemic and periodontal conditions: this issue can interfere with the effectiveness of the tested adjunctive therapy; therefore, further sub-group analyses are required. To avoid further fragmentation in the statistical analysis, sub-group analyses regarding PPD, BoP, and study design were performed, while HbA1c levels and different protocols for aPDT were not included.

In this study, HbA1c values were not homogeneously distinguished between poorly and well-controlled patients, except in one study.

Although protocols for aPDT appeared quite similar in the studies included in this review, their heterogeneity can greatly influence the antimicrobial effectiveness of the therapy [85,86]: this is particularly evident in terms of the timing, form, and number of applications (single or multiple sessions) and the technical features of procedures (wavelength, photosensitizing agent used, diameter of the fibers used, power density, and energy output).

In addition, one study did not report any statistical comparison [47]. Furthermore, among the studies evaluated, five utilized the split-mouth design, in which the responsiveness of the test and control sites can be potentially influenced by patient-related systemic and periodontal conditions.

Assessment of publication bias was not formally conducted in this meta-analysis. Although funnel plot analysis could have been performed given the number of included studies (n = 15), the substantial heterogeneity observed across outcomes (I^2^ = 82–88%) would have limited the interpretability of such an analysis. Statistical tests for publication bias, such as Egger’s test, are less reliable when significant heterogeneity is present and with fewer than 20 studies per outcome [87]. This limitation may, thus, affect the generalizability of our findings.

Finally, regarding the literature search, the choice of electronic databases of PubMed, Cochrane Library, Scopus, and Web of Science (core collection) was made according to the research question and type of information searched: these databases are known as multidisciplinary databases, providing a comprehensive search and scientific details simultaneously. Although the authors consider the search strategy satisfactory, other subject-specific databases could be included in further investigations to enlarge the search scope.

## 5. Conclusions

Within the limitations of the study, this review indicates that the non-invasive therapy of aPDT + NSPT may contribute, in the short term, to a reduction in inflammatory parameters such as PPD and BoP in patients with T2DM, indicating that this combined therapy could represent a potentially useful approach for short-term follow-up.

The recommendation to apply aPDT in T2DM patients should be considered with moderate certainty in the evidence, high heterogeneity, and methodological limitations of the analyzed trials. Although PPD and BoP can be considered reliable indicators of promising improvement in periodontal inflammation, these positive outcomes must be interpreted with caution.

## Figures and Tables

**Table 1:** General characteristics and outcomes of the included studies.

Authors	Country	Study Design	HbA1c	Groups in the Study		PPD	Parameters	Follow-Up (Months)
				Test (n)	Control (n)			
**Al-Zahrani et al. 2009** [39]	Saudi Arabia	RCT, patient assignment		SRP + aPDT (15) aPDT: non-thermal Diode Laser 670 nm, 0.01% Methylene blue for 60 s, SRP in 4 sessions.	SRP (15) SRP + doxycycline (15)	SRP only Baseline 3.24 ± 0.66; 3 months 2.64 ± 0.70; *p* = 0.001 SRP + doxycycline Baseline 3.26 ± 0.45; 3 months 2.82 ± 0.23; *p* = 0.001 SRP + aPDT Baseline 3.00 ± 0.46; 3 months 2.55 ± 0.33; *p* = 0.001	BoP Baseline (%): test 0.72 ± 0.24 control 0.72 ± 0.24 3 months test 0.54 ± 0.39 control 0.43 ± 0.28 PI Plaque Index (%) Baseline: test 0.90 ± 0.19 control 0.86 ± 0.14 3 months test 0.56 ± 0.33 control 0.59 ± 0.17 CAL Baseline (mm): test 4.33 ± 1.15 control 4.66 ± 1.32 3 months test 3.87 ± 1.16 control 4.10 ± 1.30	3
**Macedo et al. 2013** [40]	Brazil	RCT, patient assignment	>7%	SRP + aPDT (15) aPDT: Diode Laser 660 nm with Chloride Photosensitizer in a concentration of 10 mg/mL. Exposition 10 s each site. A total of 60 mW of power and power density of 28 mW/cm^2^ with an optic fiber with a diameter of 0.6 mm.	SRP (15)	Baseline Test: 2.43 ± 0.27 Control: 2.54 ± 0.53 *p* = 0.05 3 months Test: 1.82 ± 0.19 Control: 1.92 ± 0.62 *p* = 0.05	BoP Baseline (%): test 60.42 ± 12.2 control 64.5 ± 12.5 3 months test 14.7 ± 12 control 64.5 ± 12.5 PI Plaque Index (%) Baseline: test 50.24 ± 19.41 control 63.72 ± 16.92 3 months test 33.03 ± 43.52 control 45.11 ± 42.14 6 months test 29.78 ± 41.18 control 38.75 ± 40.22 CAL Baseline (mm): test 6.71 ± 1.85 control 6.63 ± 1.66 3 months test 4.95 ± 2.33 control 5.54 ± 2.19	3
**Castro dos Santos et al. 2016** [41]	Brazil	RCT, split mouth	from 6.5 to 11%	SRP + aPDT (20) aPDT: 0.005% Methylene blue for 60 s. Diode Laser irradiation for 60 s with a fiber optic of 0.6 mm. Power of 60 mW, irradiance of 2.15 W/cm^2^. Total energy delivered of 3.6 J and fluency of 1 J/cm^2^.	SRP (20)	Baseline Test: 6.15 ± 1.27 Control: 5.75 ± 0.91 *p* = 0.25 1 month Test: 4.11 ± 1.40 Control: 3.68 ± 0.89 *p* = 0.10 3 months Test: 3.78 ± 1.61 Control: 3.89 ± 0.99 *p* = 0.74 6 months Test: 3.71 ± 1.63 Control: 3.47 ± 0.97 *p* = 0.25	GI Baseline: - CAL Baseline: test 4.61 ± 1.92 control 6.35 ± 1.27 6 months test 35.4 ± 3.2 control 4.26 ± 1.30	1–3–6
**Ramos et al. 2016** [42]	Brazil	RCT, patient assignment	>7%	SRP + aPDT (15) aPDT: Phenothiazine Chloride solution 10 mg/mL for 5 min. Red Laser HELBO for 10 s for each site. A total of 70 mW of power and a power density of 28 mW/cm^2^. Optic fiber with 0.06 mm diameter, delivering a total energy of 2.79 J/cm^2^ per site.	SRP + doxycycline (15)	Baseline Test: 4.89 ± 0.68 Control: 4.86 ± 1.39 *p* = 0.05 3 months Test: 3.47 ± 0.58 Control: 2.88 ± 0.45 *p* = 0.05	BoP Baseline: test 28.13 ± 7.79 control 23.53 ± 9.33 3 months test 11.50 ± 5.41 control 13.47 ± 4.62 Plaque Index: NR CAL baseline: test 5.60 ± 0.68 control 5.53 ± 0.86 3 months test 3.90 ± 0.52 control 3.87 ± 0.52	1–3
**Barbosa et al. 2018** [43]	Brazil	RCT, patient assignment	Values below 7% measured no more than 90 days prior to selection	SRP + aPDT (6) aPDT: Methylene blue ta 10 mg/mL for 5 min. Red Diode laser 660 nm (TwinFlex). Application for 2 min. The power was 40 mW; the total energy delivered per tooth was 4.8 J.	SRP (6)	Baseline Test: 2.3 ± 0.4 Control: 2.9 ± 0.5 *p* = 0.1 1 months Test: 1.6 ± 0.2 Control: 2.0 ± 0.6 *p* = 0.1 3 months Test: 1.7 ± 0.2 Control: 2.1 ± 0.7 *p* = 0.2 6 months Test: 1.8 ± 0.3 Control: 2.2 ± 0.7 *p* = 0.3	BoP Baseline: test 43.9 ± 16.9 control 55.5 ± 33.1 3 months test 22.4 ± 8.2 control 31.7 ± 13.9 6 months test 18.7 ± 1.5 control 32.1 ± 11.2 Plaque Index Baseline: test 31.4 ± 3 control 32.4 ± 2.2 3 months test 35.4 ± 3.2 control 36.1 ± 4.6 6 months test 31.5 ± 6.2 control 35.4 ± 4.7	1–3–6
**Castro Dos Santos et al. 2019** [44]	Brazil	RCT, split mouth	from 6.5% to 11%	SRP + aPDT (19) aPDT: GaALAs (Gallium Aluminium Arsenide) Diode Laser of 809 nm; power of 1 W; for 10 s; Optic fiber of 0.06 mm	SRP (19)	Baseline Test: 6.6 ± 1.6 Control: 6.5 ± 2.0 *p* = 0.36 3 months Test: 4.4 ± 1.2 Control: 4.7 ± 1.9 *p* = 0.65 6 months Test: 3.9 ± 1.4 Control: 4.1 ± 1.5 *p* = 0.65 12 months Test: 3.6 ± 1.5 Control: 3.8 ± 1.6 *p* = 0.49	BoP: NR Plaque Index: NR CAL baseline: Δ12 months control 2.0 ± 1.8 test 2.5 ± 2.1	3–6–12
**Ivanaga et al. 2019** [45]	Brazil	RCT, split mouth	≥6.5%	SRP + aPDT (24) aPDT: Wave Diode Laser with a fiber optic of 0.06 mm in diameter, a wavelength of 600 nm, power of 0.03 W, fluency of 22 J/cm^2^, an area of 0.028 cm^2^, irradiation of 1.1 W/cm^2^, total energy of 0.6 J. Photosensitizer: NS.	SRP (24)	Baseline Test: 5.71 ± 0.92 Control: 5.67 ± 0.78 *p* > 0.05 3 months Test: 4.33 ± 1.78 Control: 4.58 ± 1.24 *p* > 0.05 6 months Test: 4.47 ± 1.40 Control: 4.70 ± 1.37 *p* > 0.05	BoP Baseline (%): test 100 control 100 3 months test 42.60 ± 44.23 control 48.26 ± 38.53 6 months test 34.99 ± 40.33 control 30.64 ± 34.50 PI Plaque Index (%) Baseline: test 68.24 ± 38.23 control 75.61 ± 32.22 3 months test 33.03 ± 43.52 control 45.11 ± 42.14 6 months test 29.78 ± 41.18 control 38.75 ± 40.22 CAL Baseline (mm): test 6.71 ± 1.85 control 6.63 ± 1.66 3 months test 4.95 ± 2.33 control 5.54 ± 2.19 6 months test 34.99 ± 40.33 control 30.64 ± 34.50	3–6
**Mirza et al. 2019** [46]	Pakistan, Saudi Arabia, Japan	RCT, patient assignment	≥6.5%	SRP + aPDT (15) aPDT: Diode Laser HELBO 670 nm, power of 150 mW, fluency of 22 J/cm^2^, and density of 1.1 W/cm^2^. Methylene blue with 0.005% concentration.	SRP (15)	Baseline Test: 3.53 ± 0.42 Control: 3.23 ± 0.61 3 months Test: 2.72 ± 0.38 (*p* value: 0.01 between baseline and 3 months) Control: 2.94 ± 0.59 (*p* value: 0.05 between baseline and 3 months) 6 months Test: 2.56 ± 0.41 (*p* value: 0.05 between baseline and 6 months) Control: 2.69 ± 0.46 (*p* value: 0.05 between baseline and 6 months)	BoP Baseline (%): test 0.82 ± 0.12 control 0.75 ± 0.22 3 months test 0.49 ± 0.16 control 0.48 ± 0.25 6 months test 0.41 ± 0.20 control 0.39 ± 0.29 PI Plaque Index (%) Baseline: test 0.91 ± 0.17 control 0.89 ± 0.16 3 months test 0.66 ± 0.19 control 0.59 ± 0.18 6 months test 0.56 ± 0.15 control 0.51 ± 0.19 CAL: NR	3–6
**Al-Zawawi et al. 2020** [47]	Saudi Arabia	RCT, patient assignment	Test Group Values at Baseline 4.3 ± 0.04 Test Group Values at 6 month’s follow-up 4.2 ± 0.1 Control Group Values at Baseline 4.3 ± 0.05 Control Group Values at 6 months’ follow up 4.2 ± 0.05	SRP + aPDT (32) aPDT: Methylene blue 0.005%. Diode Laser 660 nm and 150 mW, irradiation was for 60 s with a fiber optic of 0.03 mm diameter.	SRP (32)	Baseline Test: 5.2 ± 0.06 Control: 4.7 ± 0.1 mm 3 months Test: 4.9 ± 0.03 mm Control: 4.5 ± 0.07 mm 6 months Test: 5.0 ± 0.05 mm Control: 4.9 ± 0.1 mm	PI: Baseline Test 3.2 ± 0.08 Control 3.6 ± 0.05 3 months: Test 2.8 ± 0.1 Control 3.4 ± 0.02 6 months: Test 3.0 ± 0.05 Control 3.2 ± 0.03 GI: Baseline, Test 3.6 ± 0.02 Control 3.5 ± 0.04 3 months: Test 3.2 ± 0.04 Control 3.3 ± 0.06 6 months: Test 3.5 ± 0.05 mm Control 3.4 ± 0.04 CAL: Baseline Test 4.2 ± 0.06 mm Control 4.5 ± 0.05 mm 3 months: Test 4.2 ± 0.08 mm Control 4.5 ± 0.08 mm 6 months: Test 5.0 ± 0.05 mm Control 4.4 ± 0.006 mm	3–6
**Özberk et al. 2020** [48]	Turkey	RCT, split mouth	≥7%	SRP + aPDT (22) aPDT: GaAlAs Diode Laser at the 980 nm wavelength (CHEESETM GIGAA). Used for 15 s continuously at 0.4 W power. Energy density 0.5 J/cm^2^.	SRP (22)	Baseline Test: 4.09 ± 0.29 Control: 4.08 ± 0.44 1 months Test: 2.94 ± 0.29 Control: 3.11 ± 0.48 *p* = 0.05 3 months Test: 2.72 ± 0.42 Control: 2.99 ± 0.42 *p* = 0.05 6 months Test: 2.60 ± 0.30 Control: 2.90 ± 0.40 *p* = 0.05	GI Baseline (%): test 1.92 ± 0.17 control 1.91 ± 0.14 3 months test 0.40 ± 0.15 control 0.52 ± 0.17 6 months test 0.38 ± 0.10 control 0.48 ± 0.16 PI Plaque Index (%) Baseline: test 2.88 ± 0.68 control 2.89 ± 0.60 3 months test 0.54 ± 0.15 control 0.59 ± 0.16 6 months test 0.52 ± 0.12 control 0.54 ± 0.15 CAL Baseline (mm): test 4.52 ± 0.75 control 4.50 ± 0.65 3 months test 2.93 ± 0.36 control 3.06 ± 0.41 6 months test 2.79 ± 0.32 control 3.00 ± 0.39	1–3–6
**Al-Momani. 2021** [49]	Saudi Arabia	RCT, split mouth	DM Controlled 6–10% DM Uncontrolled >10%	SRP + aPDT DM Controlled (17) DM Uncontrolled (16) aPDT: ICG solution at a concentration of 0.5 mg/mL. Diode laser 810 nm (GmbH). Power 200 mW, total energy was 4 J for 10 s at each site.	SRP DM Controlled (17) DM Uncontrolled (16)	DM Controlled Baseline Test: 6.39 ± 0.59 Control: 6.47 ± 0.62 *p* > 0.05 3 months Test: 4.77 ± 0.68 Control: 5.58 ± 0.83 *p* < 0.05 6 months Test: 3.64 ± 0.84 Control: 4.92 ± 0.95 *p* < 0.05 DM Uncontrolled Baseline Test: 6.51 ± 0.35 Control: 6.65 ± 0.42 *p* > 0.05 3 months Test: 5.02 ± 0.52 Control: 5.79 ± 0.78 *p* < 0.05 6 months Test: 4.51 ± 0.90 Control: 5.17 ± 0.98 *p* > 0.05	BoP Baseline (%): test 46 ± 21.3 control 45.2 ± 22.7 3 months test 12.4 ± 5.5 control 23.3 ± 12.8 6 months test 11.8 ± 4.9 control 19.9 ± 7.9 PI Plaque Index (%) Baseline: test 56.1 ± 23.7 control 53.8 ± 26.8 3 months test 13.4 ± 6.3 control 11.4 ± 5.3 6 months test 12.0 ± 3.1 control 14.9 ± 6.7 CAL Baseline (mm): test 6.49 ± 0.39 control 6.62 ± 0.75 3 months test 4.93 ± 0.51 control 5.86 ± 0.86 6 months test 3.94 ± 0.74 control 5.04 ± 0.97	3–6
**Cláudio et al. 2021** [50]	Brazil	RCT, patient assignment	>7%	SRP + aPDT (16) aPDT: Indium Gallium Aluminium Phosphorus Diode laser 660 nm(InGaAP) for 50 s, totaling 157 J/cm^2^, energy of 4.7 J, 100 mW power, and an optical fiber with 0.03 cm^2^ diameter.	SRP (15)	Baseline Test: 5.22 ± 0.17 Control: 5.36 ± 0.18 *p* = 0.12 3 months Test: 4.04 ± 0.78 Control: 4.21 ± 0.46 *p* = 0.58 6 months Test: 3.92 ± 0.59 Control: 4.06 ± 0.67 *p* = 0.50	BoP Baseline (%): test 7.25 ± 7.01 control 6.33 ± 5.35 3 months test 3.90 ± 4.10 control 3.66 ± 3.86 6 months test 3.04 ± 3.65 control 3.44 ± 4.71 PI Plaque Index (%) Baseline: test 38.27 ± 14.44 control 35.55 ± 14.58 3 months test 33.36 ± 16.89 control 29.80 ± 17.52 6 months test 34.25 ± 19.24 control 30.78 ± 15.93 CAL Baseline (mm): test 3.61 ± 0.88 control 3.52 ± 0.71 3 months test 3.48 ± 0.76 control 3.27 ± 0.76 6 months test 3.51 ± 0.69 control 3.40 ± 0.80	3–6
**Sufaru et al. 2022** [51]	Romania	RCT, patient assignment	>6%	SRP + aPDT (24) aPDT: Diode Laser 810 nm (A.R.C) Indocyanine Green Powder. Laser applied continuously with a power of 0.2 W and a total energy of 12 J, around the tooth for 60 s.	SRP (25)	Baseline Test: 5.53 ± 0.24 Control: 5.54 ± 0.24 *p* > 0.05 6 months Test: 3.56 ± 0.19 Control: 4.10 ± 0.22 *p* < 0.05	BoP Baseline (%): test 68.67 ± 6.1067 control 67.76 ± 6.57 6 months test 4.21 ± 3.85 control 8.08 ± 5.09 PI Plaque Index (%) Baseline: test 80.04 ± 5.90 control 79.44 ± 6.31 6 months test 17.08 ± 5.14 control 17.72 ± 6.38 CAL Baseline (mm): test 4.50 ± 0.22 control 4.51 ± 0.20 6 months test 2.58 ± 0.19 control 3.15 ± 0.17	6
**Brinar et al. 2023** [52]	Slovenia	RCT, patient assignment	>7%	SRP + aPDT (12) aPDT: Diode Laser (Fotona) with a wavelength of 810 nm, a power of 250 mW, and the photosensitizing agent Indocyanine Green at a concentration of 1 mg/mL. A total of 10 s of irradiations at each site.	SRP (12)	Baseline Test: 3.3 ± 0.2 Control: 3.1 ± 0.2 3 months Test: 2.6 ± 0.2 Control: 2.7 ± 0.1 *p* = 0.001	FMBS Baseline (%): test 21.8 ± 4.2 control 26.6 ± 4.2 3 months test 7.1 ± 3.1 control 19.1 ± 3.1 FMPS Plaque Index (%) Baseline: test 48.4 ± 7.3 control 29.6 ± 8.5 3 months test 11.2 ± 5.2 control 15.9 ± 5.2 CAL Baseline (mm): test 3.7 ± 0.02 control 3.2 ± 0.2 3 months test 3.6 ± 0.2 control 3.2 ± 0.2	3
**Cláudio et al. 2024** [53]	Brazil	RCT, patient assignment	≥7%	SRP + aPDT + BM (15) aPDT: Methylene blue 0.1 mg/mL. InGaAlP Diode Laser 660 nm for 50 s with a total of 166 J/cm^2^, an energy of 5 J, a power of 100 mW, and an optical fiber with a 0.03 cm^2^ of diameter.	SRP (15) SRP + BM (15)	Baseline Test: 4.47 ± 0.39 Control (SRP): 4.8 ± 0.79 Control (BM): 4.70 ± 0.54 *p* = 0.4748 3 months Test: 3.54 ± 0.57 Control (SRP): 3.86 ± 1.18 Control (BM): 3.76 ± 1.00 6 months Test: 3.55 ± 0.15 Control (SRP): 3.81 ± 0.93 Control (BM): 3.87 ± 0.93	BoP Baseline (%): test 36.8 ± 10.56 control 35.6 ± 20.97 3 months test 23.33 ± 11.64 control 23.4 ± 10.62 6 months test 23.27 ± 12.94 control 20.8 ± 12.11 PI Plaque Index (%) Baseline: test 36.67 ± 16.06 control 34.2 ± 18.25 3 months test 23.8 ± 10.14 control 28.13 ± 19.42 6 months test 26.47 ± 11.96 control 19.53 ± 14.02 CAL Baseline (mm): test 5.4 ± 0.92 control 5.59 ± 1.52 3 months test 4.19 ± 1.10 control 4.71 ± 1.83 6 months test 4.17 ± 1.04 control 4.6 ± 1.52	3–6

RCT, randomized controlled trial; DM, diabetes mellitus; SRP, scaling and root planing; aPDT, antimicrobial photodynamic therapy; BM, active oxygen-releasing gel.

## Data Availability

Data are available from the corresponding authors upon reasonable requests.
